# Willingness to pay for a 4% chlorhexidine (7.1% chlorhexidine digluconate) product for umbilical cord care in rural Bangladesh: a contingency valuation study

**DOI:** 10.1186/1472-698X-13-44

**Published:** 2013-10-18

**Authors:** Patricia S Coffey, Mutsumi Metzler, Ziaul Islam, Tracey P Koehlmoos

**Affiliations:** 1 Technology Solutions Global Program, PATH, 2201 Westlake Ave, Seattle, WA, 98121, USA; 2 Centre for Equity & Health Systems, icddr,b, Mohakhali, Dhaka, 1000, Bangladesh; 3 Headquarters, United States Marine Corps, 3000 Marine Corps, Pentagon, Washington, DC, 20350, USA

**Keywords:** Chlorhexidine, Umbilical cord infection, Umbilical cord care, Willingness to pay, Bangladesh, Neonatal mortality, Contingency valuation

## Abstract

**Background:**

Recent trials in Bangladesh, Nepal, and Pakistan have shown that chlorhexidine is an effective antiseptic for umbilical cord care compared to existing community-based cord care practices. Because of the aggregate reduction in neonatal mortality in these trials, interest is high in introducing a 7.1% chlorhexidine digluconate liquid or gel that delivers 4% chlorhexidine for umbilical cord care in Bangladesh and elsewhere.

**Methods:**

In 2010, we conducted a household survey applying a contingent valuation method with 1717 eligible couples (pregnant women or women with a first child younger than 6 months old, and their husbands) in the rural subdistricts of Abhoynagar and Mirsarai in Bangladesh to assess their willingness to pay for three types of umbilical cord care products at different price points. Each respondent was asked about willingness to pay prefixed prices for any one of three 7.1% chlorhexidine digluconate products: 1) a single-dose liquid, 2) a multi-dose liquid, or 3) a gel formulation. Each also reported the maximum price they were independently willing to pay for their selected product. We compared participant willingness-to-pay responses to the prefixed prices with their independently reported maximum prices for each type of the product separately. The comparison identified to what extent the respondents’ positive responses to the prefixed prices matched their independently reported maximum prices.

**Results:**

This cross matching revealed that willingness to pay the prefixed prices was 41% for the single-dose liquid, 33% for the multi-dose liquid, and 31% for the gel formulation. Although the majority of the respondents were unwilling to pay the prefixed prices, all were willing to pay some amount and reported they could borrow money if necessary. Subsequent analysis of responses to the multi-dose liquid showed borrowing money would not be required if the unit price was Bangladeshi taka 15–25.

**Conclusions:**

A unit price of Bangladeshi taka 15–25 (US$0.21–0.35) for multi-dose 7.1% chlorhexidine digluconate liquid would be affordable to the primary target population in Bangladesh. Although a large market demand could be generated if the product were available at this price point, subsidization may be required to achieve optimal coverage, especially among poorer families.

## Background

The deadline for the Millennium Development Goals (MDG) is rapidly approaching, yet the 2010 Millennium Development Goals Report revealed that without a major push, many of the MDG targets including MDG 4 on reducing child mortality are likely to be missed in most regions [1]. A major obstacle to the progress of MDG 4 has been the inability to save the lives of neonates in many low- and middle-income countries. Reaching MDG 4 will require universal coverage with key effective, affordable interventions for newborns such as chlorhexidine for umbilical cord care. Recently, chlorhexidine for umbilical cord care has been highlighted as an “affordable, effective, but underutilized life-saving commodity” for newborn health by the UN Commission on Life-Saving Commodities for Women and Children [2].

The 1998 World Health Organization guidelines on cord care recommend dry cord care as well as the application of topical antiseptics to the cord stump in areas with high infection risk [3,4]. The recently cut umbilical cord is an entry point for bacteria that cause newborn sepsis and death. Ensuring optimal cord care at birth and in the first week of life, especially in settings with poor hygiene, is a crucial strategy to prevent life-threatening sepsis and cord infections, and avert preventable neonatal deaths. In rural Bangladesh, 80% of childbirth takes place at home under poor hygienic conditions [5]. Of these, about 60% are attended by untrained attendants; 12% by trained traditional birth attendants; and 4% by relatives, neighbors, and friends. In these settings, applying substances such as mustard oil, *Nebanol* powder (neomycin sulphate + bacitracin zinc preparation), spirit (ethyl alcohol), homeopathic medicines and *belay sindoor* (vermilion powder) are common.

Recent trials in Bangladesh [6], Nepal [7], and Pakistan [8] have shown that chlorhexidine is an effective antiseptic when used for umbilical cord care. Data from the three countries indicate that use of chlorhexidine for umbilical cord care results in an aggregate 23% reduction in neonatal mortality [9].

In Bangladesh, the majority of neonatal death are due to severe infection which includes sepsis, meningitis, pneumonia, and tetanus (20%), intrapartum-related or asphyxia (23%) and complications of preterm birth (45%) [10]. In 2010, an estimated 102000 neonatal deaths occurred Bangladesh [10].

Therefore, interest in introducing a 7.1% chlorhexidine digluconate liquid or gel that delivers 4% chlorhexidine for umbilical cord care is high. Assessing the willingness to pay (WTP) of potential users for a chlorhexidine umbilical cord care product could inform the most appropriate price point that supports sustainable supply and demand and facilitate optimal uptake such that the product would be applied to all infants born in Bangladesh within the first 24 hours of birth.

## Methods

In 2010, we conducted a household survey applying a contingent valuation method (CVM) with 1717 eligible couples in Abhoynagar and Mirsarai subdistricts in Bangladesh. Our objective was to assess their willingness to pay for three types of 7.1% chlorhexidine digluconate (which delivers 4% chlorhexidine) umbilical cord care products at different price points. The three products were 1) a single-dose liquid, 2) a multi-dose liquid, and 3) a gel preparation of 7.1% chlorhexidine digluconate.

We conducted the household survey April 2010 through July 2010. Using a structured questionnaire (Additional file 1), we interviewed eligible couples—pregnant women or women with a first-born child younger than 6 months old, and their husbands—living in the rural subdistricts of Abhoynagar and Mirsarai. Our total estimated sample size of 1700 was based on the assumption that there would be a 10% difference in the proportion of the willingness to pay for chlorhexidine products between eligible couples in the two sites, Abhoynagar and Mirsarai. We used a standard formula where n = sample size, p1 = .5 (proportion of willingness to pay in pregnant women and women with first-born child aged <6 months), p2 = .4 (proportion of willingness to pay in husbands), difference = p1-p2 = .1 with power 80% and 95% confidence level. Considering 10% attrition, a total of 425 respondents in each group (two categories of women and their husbands) were calculated for Abhoynagar and Mirsarai.

The required number of respondents for each category was randomly selected from International Centre for Diarrhoeal Disease Research, Bangladesh (icddr,b) databases, which are populated by ongoing surveillance in these two areas. Wives and husbands were interviewed separately.

Using the CVM, as explained in the following steps to assess willingness to pay [11,12], the interviewer first presented a hypothetical scenario to the respondent that included a clear description of three different products containing 7.1% chlorhexidine digluconate: single- and multi-dose liquid, and multi-dose gel. We then explained each of the product indications, benefits, advantages over alternatives, and side effects. We asked about their interest in any one type of the product, and preference for type of product (i.e., liquid or gel) and dosage regimen (i.e., single day dose or multi-day dose). If they responded positively, we reminded the respondents to take into account other products currently available and their household budget. We informed them that there was no right or wrong answer and that they could decide to reject the product at any price.

Based on the respondent’s product preference, we then initiated a bidding game by introducing the four different prefixed prices for each of the three products. The prefixed price bids for the single-dose chlorhexidine liquid were Tk. 27, 32, 35, and 30 (the international exchange rate in December 2010 was US$1 = Bangladeshi Tk. 70). For the multi-dose liquid and the gel preparation they were Tk. 45, 55, 60, and 50. Starter price bids for the liquid products—Tk. 27 for the single-dose liquid and Tk. 45 for the multi-dose liquid—were suggested by a Bangladeshi manufacturer that had produced a 7.1% chlorhexidine digluconate product for an operations research. The cost of the gel, Tk. 45, was inferred from other gel products in the marketplace (none of which are used for umbilical cord care). The subsequent increases in proposed prices for all three products (moderate, higher, lower) were determined by considering the market price of locally available alternative allopathic products for umbilical cord care such as neomycin sulphate + bacitracin zinc powder and ointment, chlorhexidine digluconate 0.5% w/w in 70% isopropanol solution, povidone iodine lotion, oral amoxicillin drops, cepharadine drops, and erythromycin drops. All of these drug products could be used for a variety of medical treatments; they are not specifically used for umbilical cord care. The price of the allopathic products being currently used for neonatal cord care ranged between Tk. 10 to Tk. 60, with the popular ones costing between Tk. 25 and Tk. 35 in the study areas.

To analyze the data collected, we cross-matched the respondents’ positive responses to the prefixed prices with their independently reported maximum price for all three products. Thus, the proportion of respondents’ willing-to-pay prices that were either less, equivalent, or more than the prefixed price(s) was identified by type of preparation they preferred. Finally, based on the respondents’ proposed coping mechanism to pay a higher price than their maximum willingness to pay, we identified a price range that was affordable for all respondents.

**Figure 1:**
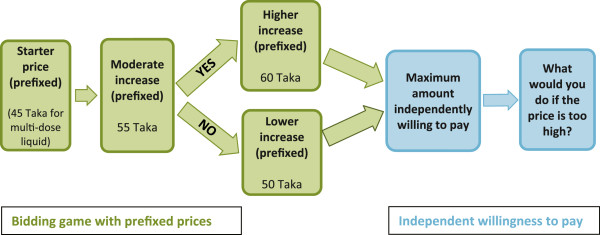
**Sequence of questions for bidding game and independent willingness to pay.** *Adapted from sequence of willingness to pay questions in Foreit and Foreit [12].

We first asked whether respondents could pay the lowest prefixed price for the product chosen and then asked about the moderate increase in price for the same. Based on respondents’ “yes” or “no” responses to the moderate increase in price, we then asked their willingness to pay either a higher or lower price. Finally, we asked an open-ended question on what would be the maximum price they would be independently willing to pay (i.e., regardless of prefixed prices assigned to each product) and how they would cope if the price was higher than their ability to afford it (Figure 1).

The Ethical Review Committee at icddr,b approved this protocol (number PR-09077). Informed written consent was obtained from each of the respondents at the beginning the interview using the approved voluntary consent form.

After editing, all quantitative data were entered into visual Fox Pro version 6.0 (Microsoft Corporation, Washington, USA) and cleaned. Univariate and bivariate analyses were done using SPSS Statistics version 13 (IBM Corporation, New York, USA) and results were presented by frequency distribution, cross tabulation, mean, and median.

## Results

We interviewed a total of 1717 respondents: 427 pregnant women, 445 women with a first-born child less than 6 months old, and 845 husbands (see Table 1 for demographic characteristics of respondents).

**Table 1:** Demographic characteristics of respondents (n = 1717)

**Variables**	**Abhoynagar %**			**Mirsarai %**		
	**n = 854**			**n = 863**		
**Age in years**	Wife n = 433	Husband n = 421	Total n = 854	Wife n = 439	Husband n = 424	Total n = 863
<20	23.1	0.0	11.8	13.8	0.2	7.2
20–24	38.2	14.6	26.7	39.1	7.4	23.7
25–29	23.3	34.7	28.9	30.5	22.7	26.7
30–34	10.8	20.3	15.4	12.0	28.7	20.1
35–39	4.1	15.6	9.7	3.8	19.9	11.6
40+	0.5	14.8	7.5	0.9	21.1	10.7
Median age in years	22	30	26	24	32	28
**Education/schooling**						
No school; can’t sign	1.4	4.3	2.8	4.1	9.3	6.6
No school; can sign	8.5	11.0	9.7	13.1	21.5	17.2
Primary school incomplete (1–5 years)	12.6	22.5	17.4	11.7	20.8	16.1
Primary school complete	11.7	9.1	10.4	8.1	6.0	7.1
Secondary school incomplete (6–10 years)	55.6	37.1	46.5	53.5	28.9	41.6
Secondary school complete	5.5	6.0	5.7	4.5	7.2	5.8
High school incomplete	0.9	2.4	1.6	2.9	0.7	1.9
High school complete	2.7	5.3	4.0	2.0	2.6	2.3
Degree/graduation complete	0.2	1.4	0.8	0.0	1.9	0.9
Other	0.9	0.9	0.9	0.0	1.0	0.5
**Occupation**						
Housewife	98.4	0.0	50.3	99.3	0.0	51.1
Farmer/agriculture	0.5	20.3	10.2	0.0	16.7	8.1
Small trade	0.0	24.6	12.0	0.0	31.6	15.3
Day laborer	0.2	15.1	7.5	0.7	10.5	5.5
Rickshaw/van puller	0.0	6.7	3.3	0.0	6.0	2.9
Hawker	0.0	0.2	0.1	0.0	1.0	0.5
Driver	0.0	2.2	1.1	0.0	5.7	2.8
Petty job	0.5	22.7	11.3	0.0	19.9	9.6
Unemployed	0.0	3.7	1.9	0.0	5.5	2.7
Other	0.5	4.3	2.3	0.0	3.1	1.5
**Number of children**	**Abhoynagar %**			**Mirsarai %**		
	**n = 854**			**n = 863**		
0	20.5			21.8		
1	37.2			32.9		
2	25.1			23.3		
3	12.6			14.1		
4+	25.1			7.9		

The proportion of respondents earning more than Tk. 5000 per month was higher in Mirsarai (77%) compared to Abhoynagar (58%). The average monthly income derived from their primary source was higher in Mirsarai (Tk. 12300 equivalent to US$176) than Abhoynagar (Tk. 7315 equivalent to US$105) and statistically significant (p < 0.05) [the international exchange rate in December 2010 was US$1 = Bangladeshi Tk. 70]. However, this difference in income did not appear to affect their willingness to pay; we did not find any correlation between income and willingness to pay (r = 0.085).

The large majority of the women (>91%) in both sites reported that they had used some type of product for drying and preventing cord infection when their last child was born. Husbands were less aware of the subject than their wives—of the husbands, 6% to 9% had no such experience, while a large proportion (42% to 49%) did not know about use of any such product.

Respondents reported that they had used various products for umbilical cord care. Use of mustard oil ranked at the top followed by *Nebanol* powder (neomycin sulphate + bacitracin zinc preparation), spirit (ethyl alcohol), homeopath medicines and *belay sindoor* (vermilion powder). Very few had used more traditional products such as cow dung, ash dust, chili, turmeric paste, or breast milk.

All respondents were interested in using 7.1% chlorhexidine digluconate and participated in the willingness-to-pay bidding game. However, their responses differed in relation to the dosage and preparation options. Although 65% (n = 1109) of the respondents preferred the gel preparation, their overall willingness to pay the prefixed prices for it was lowest (31%). The remaining 35% of the respondents (n = 608) preferred liquid preparations. The majority of respondents who preferred one of the three products were willing to pay less than the prefixed prices, but all respondents were willing to pay some amount of money for the product they preferred.

### Gel product

**Figure 2:**
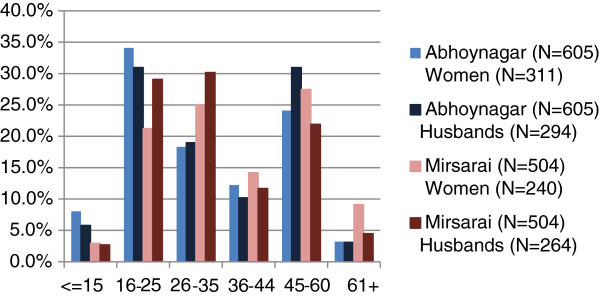
Graph representation of respondents’ WTP for chlorhexidine gel.

For the gel product, 31% of our respondents were willing to pay the equivalent of or more than the prefixed prices with 26% willing to pay prices that were equivalent to prefixed prices of Tk. 45 to Tk. 60 and 5% willing to pay more than the prefixed prices of more than Tk. 61. The remaining 69% of our respondents were willing to pay between Tk. 15 and Tk. 44 (Table 2 and Figure 2).

**Table 2:** Respondents’ WTP for chlorhexidine gel*

**Price category**	**Price in taka**	**Abhoynagar %**			**Mirsarai %**			**Overall %**	
		**n = 605**			**n = 504**			**n = 1109**	
		**PW n = 163**	**MB n = 148**	**HS n = 294**	**PW n = 108**	**MB n = 132**	**HS n = 264**		
Less than prefixed prices	<=15	5	12	6	2	3.8	3	5	69
	16–25	33	36	31	11	29.5	29	29	
	26–35	20	17	19	24	25.8	30	23	
	36–44	15	10	10	18	11.4	12	12	
Equivalent to prefixed prices	45–60	24	24	31	37	19.7	22	26	31
More than prefixed prices	61+	4	2	3	8	9.8	5	5	

*Percentages may not equal 100 due to rounding.

PW = pregnant woman; MB = mother with baby less than 6 months old; HS = husband.

### Single-dose liquid preparation

**Figure 3:**
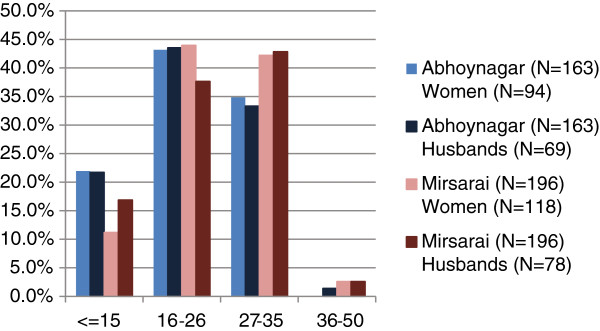
Graph representation of respondents’ WTP for single-dose chlorhexidine liquid.

For the single-dose liquid preparation, 41% of our respondents were willing to pay the equivalent of or more than the prefixed prices; 39% were willing to pay prices that were equivalent to the prefixed prices of Tk. 27 to Tk. 35, and 2% were willing to pay more than the prefixed prices of Tk. 36 to Tk. 50. The remaining 59% of our respondents were willing to pay between Tk. 15 and Tk. 26 (Table 3 and Figure 3).

**Table 3:** Respondents’ WTP for a single-dose chlorhexidine liquid*

**Price category**	**Price in taka**	**Abhoynagar %**			**Mirsarai %**			**Overall %**	
		**n = 163**			**n = 196**			**n = 359**	
		**PW n = 32**	**MB n = 62**	**HS n = 69**	**PW n = 63**	**MB n = 55**	**HS n = 78**		
Less than prefixed prices	<=15	33	16	22	15	7	17	17	59
	16–26	30	50	44	42	46	38	42	
Equivalent to prefixed prices	27–35	37	34	33	44	41	43	39	41
More than prefixed prices	36–50	0	0	1	0	6	3	2	

*Percentages may not equal 100 due to rounding.

PW = pregnant woman; MB = mother with baby less than 6 months old; HS = husband.

### Multi-dose liquid preparation

**Figure 4:**
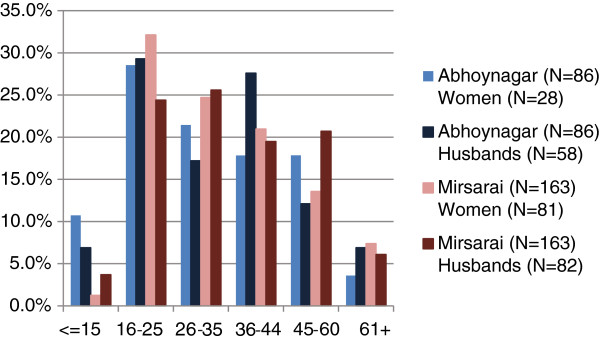
Graph representation of respondents’ WTP multi-dose chlorhexidine liquid.

For the multi-dose liquid preparation, 33% of our respondents were willing to pay the equivalent of or more than the prefixed prices; 30% were willing to pay prices that were equivalent to prefixed prices between Tk. 45 and Tk. 60, and 3% willing to pay more than the prefixed prices of more than Tk. 61. The remaining 67% of our respondents were willing to pay between Tk. 15 and Tk. 44 (Table 4 and Figure 4).

**Table 4:** Respondents’ WTP for multi-dose chlorhexidine liquid

**Price category**	**Price in taka**	**Abhoynagar %**			**Mirsarai**			**Overall %**	
		**n = 86**			**n = 163**			**n = 249**	
		**PW n = 19**	**MB n = 9**	**HS n = 58**	**PW n = 42**	**MB n = 39**	**HS n = 82**		
Less than prefixed prices	<=15	5	22	7	0	3	4	4	67
	16–25	26	33	29	26	39	24	29	
	26–35	21	22	17	26	23	26	23	
	36–44	26	0	28	19	23	20	11	
Equivalent to prefixed prices	45–60	16	22	12	19	8	21	30	33
More than prefixed prices	61+	5	0	7	10	5	6	3	

PW = pregnant woman; MB = mother with baby less than 6 months old; HS = husband.

In cases where the prefixed prices were higher than the maximum willingness to pay, the majority of our respondents reported that they would cope with the shortfall by borrowing money. However, for prices ranging from Tk. 15 to Tk. 25, respondents did not report such coping mechanism. Overall, 33% of the respondents were willing to pay Tk. 15 to Tk. 25 for the multi-dose chlorhexidine liquid, and the remaining 67% were willing to pay some amount from Tk. 25 to more than Tk. 60. It is expected that all respondents in this 67% would be willing to pay lesser prices ranging between Tk. 15 and Tk. 25 because originally they were willing to pay more.

Following cross matching, only 31% of the respondents were willing to pay the prefixed prices ranging between Tk. 45 and Tk. 60 for the gel product, 41% of the respondents were willing to pay the prefixed prices of between Tk. 27 and Tk. 35 for the single-dose liquid preparation, and only 33% of the respondents were willing to pay the prefixed prices of between Tk. 45 and Tk. 60 for the multi-dose liquid preparation. We further assessed the price implications of the multi-dose liquid preparation because it was the product used in the Bangladesh randomized controlled trial [6]. If the unit price was set at any point between Tk. 45 and Tk. 60 for this product, 67% of the respondents would need to borrow money in order to purchase it (Table 5).

**Table 5:** Price implications of a multi-dose chlorhexidine liquid

Price in taka*	**Percent willing to pay**	**Demand side implications**
15–25 (US$0.21–0.35)	33	Borrowing money is not required by any respondent interviewed.
26–35 (US$0.37–0.50)	23	Borrowing money is required for 33% of the respondents interviewed.
36–44 (US$0.51–0.63)	11	Borrowing money is required for 56% of the respondents interviewed.
**45–60** (US$0.64–0.85)	33	Borrowing money is required for 67% of the respondents interviewed.
*Total*	*100*	

*Conversion based on international exchange rates as of December 2010: US$1 = Bangladeshi Tk. 70.

## Discussion

Given the pattern of willingness-to-pay responses and the proposed coping mechanisms to higher prices, this study found that the unit price of a 7.1% chlorhexidine digluconate product (delivering 4% chlorhexidine) should range between Tk. 15 and Tk. 25 for it to be accessible.

The majority of the respondents were not willing to pay the prefixed prices, but everyone was willing to pay some amount of money. This could be because the prefixed prices we used were too high. However, for the sake of preventing newborn cord infections, the majority of our respondents were willing to cope with higher prices by borrowing money. This willingness to pay for a product that would prevent infection in their newborn was very similar across all respondent groups. Although income level has been positively associated with the likelihood of a doctor’s visit for a sick child [13], our findings suggest that behavior related to preventive medicine may be different. Interestingly, we did find one gender differential in willingness to pay where husbands reported being willing to pay more in Mirsarai compared to Abhoynagar for multi-dose liquid product.

The most popular product that we introduced into this willingness-to-pay study was the gel. Although this particular product was not used in any of the randomized controlled trials performed in Nepal, Bangladesh, and Pakistan, it is currently being scaled up for general use in Nepal. Results from a hospital-based randomized non-inferiority trial of chlorhexidine gel and liquid suggest that satisfaction and compliance were high for both products and that the gel formulation was not inferior to the liquid [14]. For this reason, we chose to include gel in the current study as the gel could be an option for Bangladesh in the future, subject to increasing production capability of the local manufacturer. The preference for chlorhexidine gel demonstrated by a large majority of the study participants is indicative of impending demand for the gel product.

The greatest challenge to the contingent valuation method is whether responses to hypothetical questions were representative of respondents’ actual payment behavior when faced with reality (hypothetical versus actual payment), or how well did hypothetical WTP predict what people were actually willing to pay. For assessing validity of such hypothetical WTP responses, a simulated market experiment [15] is necessary where hypothetical WTP is compared with actual purchase decision. However, we could not do such experiment for the product of interest was not commercially available in the local market at the time this survey. Further study limitations may reflect starting point bias in the bidding game used. While we tried to reflect accurate pricing as much as possible, it is likely that actual pricing will be different when the product is eventually released on the market in Bangladesh. This starting point bias may be especially important if the pricing for any future chlorhexidine product is not flexible [16].

In addition to study setting, other factors that may have influenced the participants in making their choice of product and price options could be their experience of using other products including price, knowledge and practice of prevention and treatment of cord infection. Most of the respondents were familiar with some symptoms of cord infection and types of indigenous and allopathic preparation used for cord care. This familiarity with the condition of interest and currently available products has enhanced the understanding of the hypothetical scenario related to valuation of 7.1% chlorhexidine digluconate products.

## Conclusions

A unit price of Tk. 15 to Tk. 25 (US$0.21 to US$0.35) for multi-dose 7.1% chlorhexidine (delivering 4% chlorhexidine liquid would be affordable to the primary target population in Bangladesh. Although large market demand could be generated if the product were made available at this price point, subsidization may be required to achieve optimal coverage. A subsidization strategy would be especially important to reach poorer mothers since those families that have home births often make out-of-pocket payments to receive care. Increasing availability and accessibility to chlorhexidine for umbilical cord care may provide an avenue for attaining MDG 4 through a reduction in neonatal mortality.

## Abbreviations

Tk: Bangladeshi taka; CVM: Contingent valuation method; icddr,b: International Centre for Diarrhoeal Disease Research, Bangladesh; MDG: Millennium development goals; PATH: Program for Appropriate Technology in Health; USAID: United States Agency for International Development; WTP: Willingness to pay.

## Competing interests

The authors declare that they have no competing interests.

## Authors’ contributions

PC, MM, and TK developed the concept and design for the study; ZI acted as Principal Investigator, and conducted field research and data analysis; all authors drafted, read, and approved the final manuscript.

## Pre-publication history

The pre-publication history for this paper can be accessed here:

http://www.biomedcentral.com/1472-698X/13/44/prepub

## Supplementary Material

Additional file 1 Questionnaire for willingness to pay for 4% chlorhexidine products in rural Bangladesh. Click here for file
