# Supplementary material for: Willingness to pay for a 4% chlorhexidine (7.1% chlorhexidine digluconate) product for umbilical cord care in rural Bangladesh: a contingency valuation study
Source: BMC Int Health Hum Rights. 2013 Oct 18;13:44. doi: 10.1186/1472-698X-13-44 (PMC4016526; doi:10.1186/1472-698X-13-44)
Supplement: Additional file 1 — Questionnaire for willingness to pay for 4% chlorhexidine products in rural Bangladesh. [file 1472-698X-13-44-S1.docx]

**Questionnaire for Willingness to Pay for
4% Chlorhexidine Products in Rural Bangladesh**

**Household survey**

*(English translation)*

###### Face Sheet

Household ID/Current ID:

Questionnaire #: ______________

Name of interviewer: _______________________________

Date of interview: ________________________ Time start: _______________________

## Section-1: General information (to be collected from the surveillance records).

1. Name of the respondent: ___________________________ b. Sex: Male 🞎 Female 🞎
2. Category of respondent: i) Currently pregnant, ii) Woman with <6-month child,

iii) Husband

1. Respondent’s address: Village ____________________ Union ____________________

Upazila ____________________

1. Name of the spouse: _______________________________
2. Name of household head: ___________________________ Sex: Male 🞎 Female 🞎

**Note for interviewer**: Please greet the respondent gently and introduce yourself. Say a few words about the purpose of your visit. Read out and explain clearly what is written in the consent paper. After obtaining written consent of the respondent, proceed to interview saying the following words:

“I will start with some general questions about you and your family and then we will talk about how you practice home delivery and newborn care and finally about your interest and willingness to pay for “a new product” (an antiseptic medicine named 4% chlorhexidine for topical use) that protects neonates from umbilical cord infection.”

## Section-2: Respondent’s socio-economic background.

| **Sl. #** | **Questions** | Responses and codes | Skip |
| --- | --- | --- | --- |
| 1. | How old are you (on your last birthday)? | __________years |  |
| 2. | What is your marital status? | Married/in union 1  Separated/deserted 2  Divorced 3  Widowed 4 |  |
| 3. | How many times have you gotten married? | Once 1  Twice 2  Thrice 3 |  |
| 4. | How many living children do you have? | Biological ____# of children 1  Stepchildren ____# 2  Adopted ____# 3 |  |
| 5. | What is your educational background? | No formal education 1  Primary school incomplete 2  Primary school (1–5 grade) completed 3  Secondary school (6–10) incomplete 4  Secondary school completed 5  Higher secondary (11–12) incomplete 6  Higher secondary completed 7  Bachelor degree 8  Others (specify) 77 |  |
| 6. | What is your main occupation? | Housewife 1  Farmer 2  Small trader (specify) 3  Day laborer 4  Rickshaw/van puller 5  Vender/hawker 6  Driver (specify) 7  Job (specify) 8  Unemployed 9  Others (specify) 77 |  |
| 7. | How much do you earn per month from your main occupation? | ­­­­­­­­­­­­­­­­____________Tk/month |  |
| 8. | Do you do any other income-generating activity/have source of income in addition to your main occupation that you just mentioned? | Yes 1  No 2 |  |
| 9. | What do you do/what is that additional source? | Specify: ______________________________ |  |
| 10. | How much do you earn per month from this additional source? | ____________Tk/month |  |
| 11. | Does anyone else provide monetary support to your family?  *(Multiple responses acceptable)* | None 1  Husband/wife 3  Father/mother 4  Children 5  Others (specify) 77 |  |
| 12. | What is your family’s total monthly income? | ___________Tk/month  Don’t know 99 |  |
| 13. | How much did you spend last month on health care for yourself, your spouse, and children? | Self____spouse____kids____=______total Tk |  |
| 14. | What is your family’s total monthly expenditure (including food, utility, education, health, clothing, toiletries, etc.)? | ___________Tk last month  Don’t know 99 |  |
| 15. | In your family, who actually makes decisions about spending for health care? | Myself 1  My husband/wife 2  Jointly (specify) 3  Father/mother 4  Father-in-law/mother-in-law 5  Others (specify) 77 |  |
| 16. | Did you receive any micro credit from any source during the last year? | Yes (specify source) 1  No 2 |  |
| 17. | What did you do with that money? | Specify: __________________ |  |
| 18. | Does your household own cultivable land? | Yes 1  No 2 |  |
| 19. | How much cultivable land do you possess now? | _______________(quantify) |  |

## Section-3: Knowledge and experience of home delivery and newborn care.

| **Sl. #** | **Questions** | **Responses and codes** | **Skip** |
| --- | --- | --- | --- |
| 20. | Is it your (your wife’s) first pregnancy during current wedlock?  *(Your wife that we interviewed)* | Yes 1  No 2 | If response is code 1, then skip questions 21 through 28 and start again with question 29. |
| 21. | How many times have you (your wife) gotten pregnant?  *(Your wife that we interviewed, inclusive of current or last pregnancies, as the case may be)* | Two times 1  Three times 2  Four times 3 |  |
| 22. | What was the pregnancy outcome last time?  *(Outcomes include live birth, twin, still birth, abortion, neonatal death)* | Single live birth 1  Single still birth 2  Twin live birth 3  Twin still birth 4  One live, one still 5  Abortion 6  Neonatal death 7 | If response is code 7, ask question 23. If response was not code 7, skip question 23. |
| 23. | What was the cause of death? | Cause: _______________________________  Don’t know 99 |  |
| 24. | Where did you go for delivery last time? | Home delivery 1  Upazila health complex 2  MCWC 3  District hospital 4  Private clinic 5  Other (specify) 77  Not applicable 88 | If response is code 88 skip the next questions. |
| 25. | Who assisted your (your wife’s) delivery last time? | TBA 1  Female relatives/neighbors 2  FWV 3  SBA 4  Female nurse 5  Others (specify) 77 |  |
| 26. | Did you then use (or advised to use) anything to protect your child’s umbilicus from infection? | Yes 1  No 2 |  |
| 27. | If yes, what did you put on the umbilicus? | Name of the product: ____________________ |  |
| 28. | Who advised you to use this? |  |  |
| 29. | Where do you prefer your (your wife’s) delivery to take place?  *(Do not probe)* |  |  |
| 30. | Whom do you prefer to assist your (your wife’s) delivery?  *(Do not probe)* |  |  |
| 31. | Please tell me what should you do for your newborn baby immediately after delivery?  *(Do not probe)* | Start exclusive breast feeding 1  Care of umbilical cord 2  Maintain cleanliness 3  Immunization 4  Keep baby warm 5  Give eye care 6  Give vitamin K injection 7  Others (specify) 77 |  |
| 32. | Please tell me, what are the common diseases that might affect your newborn child?  *(Do not probe)* | Pneumonia 1  Umbilical infection 2  Diarrhoea 3  Malnutrition 4  Common cold 5  Others (specify) 77 |  |
| 33. | Do you know how to protect the umbilical cord from infection? | Yes 1  No 2 |  |
| 34. | How would you do that for your neonate? |  |  |
| 35. | Do you know what the signs of umbilical cord infection are?  *(Do not probe)* | Signs:________________________________ |  |
| 36. | If you observe any of these signs, where would you go?  *(Do not probe)* | Will go to private MBBS doctor 1  Will go to pharmacy 2  Will go to government hospital 3  Will go to village doctor 4  Will go to homeopath 5  Will go to NGO clinic/worker 6  Others (specify) 77 |  |

## Section-4: Contingent valuation scenario for assessing willingness to pay.

**Note for interviewer:** Please speak to the respondent precisely what is written in the following paragraph. It is essential to clearly articulate and portray what the “product” (4% chlorhexidine preparation) is about; its presentation, use, and benefits for newborn; and importance of consumer’s response for setting price. This survey neither promotes the product commercially nor discourages the use of alternatives, if any, but only intends to gather evidence of willingness to pay of eligible couples residing in rural areas for 4% chlorhexidine preparations who prefer home delivery. Please record the time required to make respondent understand the product description.

“You might know that the umbilical cord that maintains blood circulation between mother and baby during intrauterine life is cut and tied at the neonatal end soon after delivery of the baby. As a result the cut end (cord stump) lying on the neonate’s abdomen is naturally exposed to the external environment and remains susceptible to infection if an antiseptic is not used and cleanliness is not maintained. This risk of getting infection is much higher in home delivery settings where hygienic conditions are often not maintained properly during and after delivery. You might know that the majority of child births take place at home in our country attended by unskilled attendants—for which this concern is very high in our context. Inadequate hand washing of the birth attendant/caregivers before cord cutting, use of unclean blade/knife and suture material, and wiping and wrapping of the baby by unclean clothing can easily infect the cut end of the cord. Even after complete separation of the umbilical cord remnant from the newborn’s body, the point of cord attachment remains vulnerable to infection for some time until that area becomes completely dry. The umbilicus may get infected at any point of time between cord cutting and natural drying due to unhygienic practices of the caregivers and can quickly spread into the newborn’s blood stream causing fatal sepsis (septicemia) leading to neonatal death. This is one of the important causes of the high rate of neonatal mortality in Bangladesh.

Such unwanted neonatal deaths can be easily prevented by using this simple product ***[Interviewer: please show the picture of the product to the respondent]*** —4% chlorhexidine preparations. Community-based studies in Nepal have shown clear evidence of effectiveness of this product in preventing umbilical infection in neonates born at home. A similar experiment is near completion in parts of Bangladesh as well. We hope this antiseptic will be available soon in liquid or gel form in Bangladesh. It is to be applied locally on and around the newborn’s umbilicus only once (single dose) or once daily for 7 days (multiple dose) after cord cutting. Use in adequate dosage will ensure the safety of your child’s umbilicus from getting infection and thereby prevention of unwanted outcomes.

PATH, a nonprofit, international organization that works for better health globally, is working in collaboration with a local drug manufacturer to make this product available in Bangladesh. ICDDR,B is collaborating with this partner organization in conducting this demand assessment survey only. For introduction of this product, we need to assess its demand among potential users like you. Based on the above-mentioned information, our aim is to assess your interest in the product, and willingness and ability to pay for it in the future. We are interested in understanding whether the price we are asking for would be acceptable, too high or too low for you, how you might be affected by this price, and what factors might affect your choice.

We need your full cooperation in generating correct and complete information with regard to your willingness and ability to pay. I will mention a particular price and will ask your response followed by questions on your reasons for agreeing or disagreeing to a particular price, the maximum amount you are willing to pay (irrespective of what we would ask for) and what would you do if the price goes beyond your affordability. Kindly be reminded that you may have other choices or substitutes that you may consider. Please imagine that your income would remain the same over the projected period and you have other household expenses to bear. It is acceptable not to want this product and therefore not be willing to pay any amount. Please know that there is no right or wrong answer.”

37. Are you interested in using 4% chlorhexidine product if advised for your newborn child?

-Yes (skip question 38 and continue interview) 1

-No (interview will be stopped by gently asking question 38 only) 2

38. May I know why you are not interested in using the 4% chlorhexidine product?

Reason(s): _______________________________________________________________

**[Interviewer: Please note the reasons, say thanks to the respondent, and leave the place for next respondent]**

| **Sl. #** | **Questions** | **Responses and codes** | **Skip** |
| --- | --- | --- | --- |
| 39. | Suppose 4% chlorhexidine is available in the market as a liquid and a gel preparation. Which one would you prefer for your child? | Liquid preparation 1  Gel preparation 2 | If response is code 1, go to question 40.  If response is code 2, go to question 49. |
| 40. | Suppose the liquid preparation of chlorhexidine is available for single-dose application (10 ml) for 1 day soon after cutting the cord and multiple-dose application (60 ml) for 7 days after cutting the cord. Which one would you prefer for your child and why? | Single dose (1 day) 1  Multiple doses (7 days) 2  Reasons______________________________ | If response is code 1, go to question 45 (complete 45–48 and then 53–56).  If response is code 2, go to question 41 (complete 41–44 and then 53–56). |
| 41. | Suppose the price of multiple-dose 4% chlorhexidine solution in a 60-ml container is Tk 45. Would you be willing to buy? | Yes 1  No 2 |  |
| 42. | Suppose the price of multiple-dose 4% chlorhexidine solution in a 60-ml container is Tk 55. Would you be willing to buy? | Yes 1  No 2 |  |
| 43. | Suppose the price of multiple-dose 4% chlorhexidine solution in a 60-ml container is Tk 60. Would you be willing to buy? | Yes 1  No 2 |  |
| 44 | Suppose the price of multiple-dose 4% chlorhexidine solution in a 60-ml container is Tk 50. Would you be willing to buy? | Yes 1  No 2 |  |
| 45. | Suppose the price of single-dose 4% chlorhexidine solution in a 10-ml container is Tk 27. Would you be willing to buy? | Yes 1  No 2 |  |
| 46. | Suppose the price of single-dose 4% chlorhexidine solution in a 10-ml container is Tk 32. Would you be willing to buy? | Yes 1  No 2 |  |
| 47. | Suppose the price of single-dose 4% chlorhexidine solution in a 10-ml container is Tk 35. Would you be willing to buy? | Yes 1  No 2 |  |
| 48. | Suppose the price of single-dose 4% chlorhexidine solution in a 10-ml container is Tk 30. Would you be willing to buy? | Yes 1  No 2 |  |
| 49. | Suppose the price of 10 g of 4% chlorhexidine gel is Tk 45. Would you be willing to buy? | Yes 1  No 2 |  |
| 50. | Suppose the price of 10 g of 4% chlorhexidine gel is Tk 55. Would you be willing to buy? | Yes 1  No 2 |  |
| 51. | Suppose the price of 10 g of 4% chlorhexidine gel is Tk 60. Would you be willing to buy? | Yes 1  No 2 |  |
| 52. | Suppose the price of 10 g of 4% chlorhexidine gel is Tk 50. Would you be willing to buy? | Yes 1  No 2 |  |
| **[Interviewer: Please ask the remaining questions of all respondents, regardless of preparation choice.]** | | | |
| 53. | Please explain your reasons for agreeing to pay?  (*Probe the responses; multiple responses are allowed*) | Price is affordable/not high 1  Want to follow expert opinion 2  I don’t want my baby to suffer 3  Complication may cause higher cost 4  Others (specify) 77 |  |
| 54. | Please explain your reasons for not agreeing to pay?  (*Probe the responses; multiple responses are allowed*) | I can’t afford/price is too high 1  There are cheaper products 2  Others (specify) 77 |  |
| 55. | What is the maximum amount you would be willing to pay irrespective of what we’ve asked for? | _____________Tk |  |
| 56. | What would you do if the price goes beyond your ability and is too high for you? | Will look for alternatives 1  Will go to government hospital to get free 2  Others (specify) 77 |  |

**[Interviewer]**

“We’ve finished our interview. Thank you very much for your kind cooperation.”

­

Time end: ____________________

Signature of the interviewer: ___________________________________ Date: _____________________
